# Coronal alignment does not enable to predict the degree of femoral and tibial torsion

**DOI:** 10.1002/jeo2.70073

**Published:** 2025-01-20

**Authors:** Leonard Grünwald, Sophie Schmidt, Marc‐Daniel Ahrend, Tina Histing, Stefan Döbele

**Affiliations:** ^1^ Department of Trauma Surgery, BG Trauma Center Tuebingen University of Tuebingen Tuebingen Germany; ^2^ Osteotomie Komitee der Deutschen Knie Gesellschaft (DKG) Munich Germany; ^3^ Sportmedizin, Universitätsklinik Tuebingen Tuebingen Germany

**Keywords:** coronal alignment, femoral torsion, mLDFA, mMPTA, tibial torsion

## Abstract

**Purpose:**

Malalignment of the lower extremity can affect one, two or all three anatomic planes. We hypothesized an influence between the malalignment of the coronal and axial planes.

**Methods:**

A total of 356 lower extremities of 226 patients were included. Femoral and tibial torsion were assessed in computer tomographic scans while frontal plane alignment was measured in long‐leg standing radiographs. The mechanical angles were for knee phenotyping according to the coronal plane alignment of the knee classification. The correlation between the coronal alignment and torsional profile was analyzed. The population was divided into three groups according to leg alignment (valgus, neutral, varus) and gender.

**Results:**

As the coronal alignment changed from valgus to varus the tibial external torsion increased (*r* = 0.35; *p* < 0.001). Femoral internal torsion increased as well but only in the male subgroup (*r* = −0.34; *p* < 0.001). Both femoral internal torsion and tibial external torsion increased with higher mechanical lateral distal femoral angle (mLDFA) but were not related to mechanical medial proximal tibial angle. A distinct pattern of results concerning knee phenotypes in relation to femoral and tibial torsion was found.

**Conclusion:**

Tibial torsion correlated with increasing varus alignment while both femoral and tibial torsion correlated with higher mLDFA, but the correlations were weak. Therefore, the coronal alignment does not enable to predict the degree of femoral and tibial torsion. This study demonstrates that an individual approach to each patient with lower limb malalignment is unavoidable.

**Level of Evidence:**

Level III.

**Abbreviations**
ANOVA: one‐way analysis of variance
CPAK: classification of knee phenotypes according to Coronal Plane Alignment
CT scans: computer tomographic scans
HQ: high quality
JLO: joint line obliquity
mFA‐mTA: mechanical tibiofemoral angle
mLDFA: mechanical lateral distal femoral angle
mLDTA: mechanical lateral distal tibial angle
mLPFA: mechanical lateral proximal femoral angle
mMPTA: mechanical medial proximal tibial angle
OA: osteoarthritis
SD: standard deviation
ULD: ultra‐low dose

## INTRODUCTION

Malalignment of the lower extremity is known to be responsible for multiple pathologic conditions of the knee: Coronal malalignment such as varus and valgus deformity increases the risk of both the development and progression of knee osteoarthritis (OA) [2, 9, 11, 12, 28]. It is even considered to be the strongest identified risk factor for the progression of tibiofemoral and patellofemoral OA [16]. Reduced femoral internal torsion increases the contact pressure in the medial compartment of the knee [18, 25] and is associated with knee OA [8]. Increased torsional malalignment of the femur can lead to in‐toeing gait [5] as well as anterior knee pain, patellofemoral instability and recurring patella dislocation due to changes in the patellofemoral kinematics [6, 7, 10, 13, 26, 29].

To be able to treat or even prevent these pathologies, a correct radiographic assessment of the lower extremity alignment is of utmost importance. A thorough understanding of the leg alignment in the different anatomical planes makes adequate three‐dimensional analysis easier. That is why the relationship between the coronal alignment and the torsional profile must be studied. A few studies on this topic already exist but they showed inconsistent results and focused solely on patients with knee OA [3, 19, 20, 23]. Hence, the present study aimed to explore whether the degree of femoral and tibial torsion in a young German population with little to no knee OA is related to the coronal leg alignment. It was also set to investigate the correlation between the joint angles introduced by Paley [24] and the torsional profile of the lower extremities. If there was a strong correlation in terms of the axial and coronal alignment of the lower extremity, one might be able to extrapolate from the alignment in one plane to the alignment in the other plane. Furthermore, the long‐leg radiographs may contain indications that suggest doing a torsion analysis.

The authors assume that there is maybe a combination of angle values in the coronary plane that indicates a risk for increased internal or external torsion for which it is, therefore, advisable to supplement a torsion analysis.

The authors hypothesized that there would be a significant correlation between the coronal alignment defined by Paley [24] and the femoral and tibial torsion. It was also hypothesized that the rotational profile differed significantly between lower extremities with valgus, neutral and varus alignment and between genders.

## MATERIALS AND METHODS

### Study population

This retrospective study included 356 knees of 226 patients who were treated in a German Level 1 trauma centre from April 2011 to October 2020. All patients were included if both a computer tomographic scan (CT scan) suitable for assessing femoral and tibial torsion and a long‐leg radiograph taken in <3 months were available. Only legs that underwent surgery or had a history of bone fracture as well as legs with incomplete imaging were excluded.

The mean age was 35.6 ± 14.9 years. The study population consisted of 188 (52.8%) left and 168 (47.2%) right legs: 159 (44.7%) legs belonged to male and 197 (55.3%) to female patients. Moreover, 43.8% of the knees had no OA, another 50% had only doubtful (Kellgren and Lawrence grade 1) and 6.2% had minimal (Kellgren and Lawrence grade 2) OA. No knees with severe OA (Kellgren and Lawrence grades 3 and 4) were included in the present study.

Patients were divided into three groups according to the mechanical tibiofemoral angle (mFA‐mTA): valgus group with mFA‐mTA <−1° (*n* = 113), neutral group with mFA‐mTA from −1° to 1.5° (*n* = 115) and varus group with mFA‐mTA >1.5° (*n* = 128).

### Radiographs

No radiographic images were taken for the purpose of this study.

Long‐leg radiographs were obtained using a 30/120 grid cassette with a source‐to‐image distance of 300 cm. The X‐ray beams were centred on the knees and a 25 mm steel ball was used to calibrate the images. In accordance with Paley [24], the patella was facing forward regardless of the foot position.

CT scans were acquired using a 128‐slice, single‐source CT (SOMATOM Definition Edge, Siemens Healthineers) using two different scanning protocols. Until July 2019, a high‐quality protocol was used, while in 2019, a new ultra‐low dose protocol was introduced. Both protocols are described by Keller et al. [17].

### Measurements

All measurements were performed using the 3D Knee Version 2.5.33.14489 by MediCAD (Hectec) by a radiologically fellowship trained surgeon.

Long‐leg radiographs were measured according to the method described by Paley [24]. The mFA‐mTA was defined as the angle between the mechanical axes of the femur and tibia. The mechanical axes were defined as the lines connecting the centre of the proximal and distal joints. A negative angle indicated valgus alignment.

In addition, the mechanical lateral proximal femoral angle (mLPFA) was defined as the lateral angle between the proximal joint line and the mechanical axis of the femur. The mechanical lateral distal femoral angle (mLDFA) was measured as the lateral angle between the distal femoral joint line and the mechanical axis of the femur whereas the mechanical medial proximal tibial angle (mMPTA) described the medial angle between the proximal tibial joint line and the tibial mechanical axis. The mechanical lateral distal tibial angle (mLDTA) was defined as the lateral angle between the joint line of the distal tibia and the mechanical tibial axis.

Femoral and tibial torsion were assessed by measuring the angles between the proximal and distal transversal axes of the femur and tibia as described by Waidelich et al. [30]. The proximal femoral axis was defined as a line passing through the centre of the femoral head and the centre of the greater trochanter. The latter was graphically approximated using an ellipse. A line connecting the posterior margins of the femoral condyles posed as the distal femoral axis. The proximal tibial axis was established as the line connecting the posterior cortices of the tibial condyles. Finally, the distal tibial axis was defined as a line connecting the centre of the medial malleolus and the centre of the incisura fibularis. A negative angle indicates internal torsion; a positive angle indicates retrotorsion.

To clarify the nomenclature: the mathematical label ‘‐’ stands for internal torsion terms antetorsion. Vice versa, external terms retrotorsion is marked with the mathematical label ‘+’.

Based on constitutional alignment and joint line obliquity, the knees were classified according to the coronal plane alignment of the knee (CPAK) classification [21] by an automated SPSS script.

### Statistical analysis

The statistical analysis was conducted using IBM SPSS version 28.0.1.1 for Windows. Visual examination of histograms was conducted to determine the normal distribution of the data. The significance level for all statistical tests was 0.05. The characteristics of the study population were summarized using descriptive statistics. Pearson's correlation analysis was used to evaluate the statistical significance of the relationship between the coronal alignment and the torsional profile of the lower extremity. Since the mLDTA was not normally distributed, Spearman correlation analysis was used instead.

One‐way analysis of variance (ANOVA) was implemented to determine the statistical significance of the differences between the three groups concerning the axial alignment. In addition, a post hoc analysis was executed using the Bonferroni method.

Differences between male and female lower extremities in terms of measured parameters were analyzed using the student's *t* test (Mann–Whitney U test for the mLDTA).

This study was approved by the local ethical committee (695/2020BO2).

## RESULTS

Measured parameters for coronal and axial alignment are summarized in Table 1.

**Table 1:** Coronal and torsional parameters in mean and SD.

	Mean (SD)
Femoral torsion (°)	−28.6 (10.8)
Tibial torsion (°)	34.9 (9.2)
mFA‐mTA (°)	0.5 (3.2)
mLPFA (°)	88.4 (5.4)
mLDFA (°)	87.8 (2.5)
mMPTA (°)	87.9 (2.4)
mLDTA (°)	86.9 (4.4)

Abbreviations: mFA‐mTA, mechanical tibiofemoral angle; mLDFA, mechanical lateral distal femoral angle; mLDTA, mechanical lateral distal tibial angle; mLPFA, mechanical lateral proximal femoral angle; mMPTA, mechanical medial proximal tibial angle; SD, standard deviation.

As the coronal alignment changed from valgus to varus, the femoral internal and the tibial external torsion increased (FT: *r* = −0.17, *p* = 0.001; TT: *r* = 0.35, *p* < 0.001). Although the subgroup analysis showed that a significant relationship between mFA‐mTA and femoral torsion was only present in the male subgroup (male: *r* = −0.34, *p* < 0.001; female: *r* = −0.13, *p* = n.s.).

**Figure 1:**
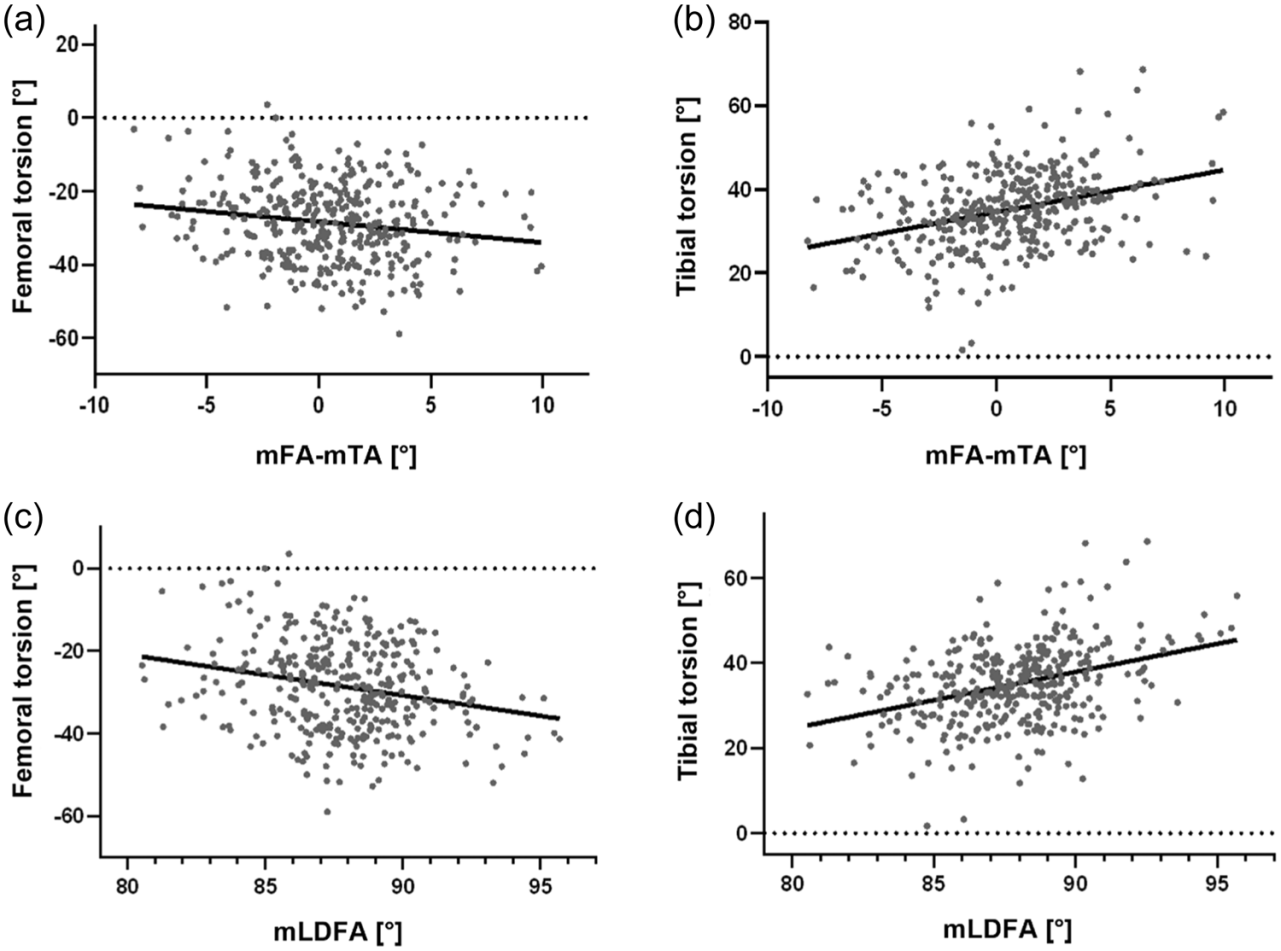
(a) Relationship between femoral torsion and coronal alignment. (b) Relationship between tibial torsion and coronal alignment. (c) Relationship between femoral torsion and mechanical lateral distal femoral angle (mLDFA). (d) Relationship between tibial torsion and mLDFA; for the torsional parameters, positive values represent external torsion, concerning the coronal alignment positive values indicate varus alignment.

Femoral internal and tibial external torsion also increased with higher mLDFA (FT: *r* = −0.23, *p* < 0.001; TT: *r* = 0.36, *p* < 0.001). These relationships are visualized in Figure 1.

A higher mLPFA was associated with increased femoral external torsion (*r* = 0.19, *p* < 0.001), while there was no connection between mLPFA and tibial torsion (*r* = −0.02, *p *= n.s.).

No correlation was observed between mMPTA and femoral or tibial torsion (FT: *r* = −0.03, *p* = n.s.; TT: *r* = −0.01, *p* = n.s.). With higher mLDTA, the femoral internal torsion increased (*r* = −0.15, *p* = 0.004) and the tibial torsion decreased (*r* = −0.24, *p* < 0.001).

There were significant differences in terms of the femoral and tibial torsion, the mFA‐mTA, the mLDFA and the mMPTA between the valgus, neutral and varus groups as presented in Table 2. The post hoc analysis showed that the femoral torsion did not differ significantly between the neutral and the varus group, but the tibial torsion, mFA‐mTA, mLDFA and mMPTA differed significantly between all three groups. There were no overall significant differences concerning mLPFA and mLDTA between any of the groups.

**Table 2:** Coronal and rotational parameters according to leg alignment; measured parameters are displayed as mean and SD; F value of the used ANOVA.

	Valgus	Neutral	Varus	F	*p* Value
Age (years)	31.9 (13.7)	33.9 (13.4)	36.1 (14.8)	2.696	n.s.
Femoral torsion (°)	−25.9 (11.7)	−29.4 (9.7)	−30.2 (10.8)	5.067	**0.007**
Tibial torsion (°)	30.9 (8.7)	35.3 (8.4)	38.3 (9.0)	21.150	**<0.001**
mFA‐mTA (°)	−3.0 (1.8)	0.3 (0.7)	3.7 (1.9)	555.500	**<0.001**
mLPFA (°)	88.2 (5.5)	88.3 (4.7)	88.7 (5.9)	0.290	n.s.
mLDFA (°)	86.0 (2.4)	88.1 (1.9)	89.2 (2.2)	64.662	**<0.001**
mMPTA (°)	89.1 (2.2)	88.1 (1.9)	86.7 (2.3)	37.725	**<0.001**
mLDTA (°)	86.6 (5.2)	87.1 (3.9)	87.2 (4.2)	0.701	n.s.

*Note*: Bold values represent statistically significant results.

Abbreviations: ANOVA, analysis of variance; mFA‐mTA, mechanical tibiofemoral angle; mLDFA, mechanical lateral distal femoral angle; mLDTA, mechanical lateral distal tibial angle; mLPFA, mechanical lateral proximal femoral angle; mMPTA, mechanical medial proximal tibial angle; n.s., not significant; SD, standard deviation.

**Figure 2:**
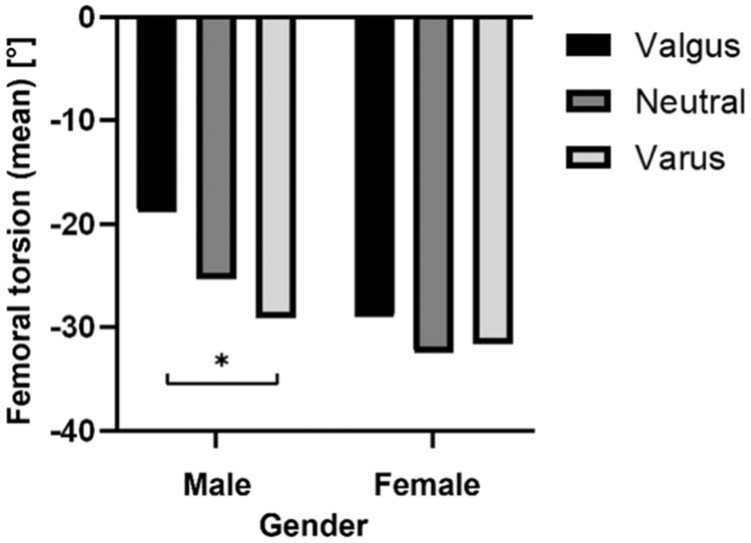
Femoral torsion according to gender and the coronal alignment group. *p < 0.001.

Further subgroup analysis showed that the difference in femoral torsion between the coronal alignment groups was significant in males only and solely between the valgus and varus groups (*p* < 0.001) as displayed in Figure 2. For the male subgroup, the mean femoral internal torsion was −18.9 ± 11.2° in the valgus group (*n* = 34), −25.4 ± 8.5° in the neutral group (*n* = 50) and −29.1 ± 10.7° in the varus group (*n* = 75). Whereas in the female group, the mean femoral internal torsion was −29.1 ± 10.2° in the valgus group (*n* = 79), −32.5 ± 9.5° in the neutral group (*n* = 65), −31.6 ± 10.9° in the varus group (n = 53) and did not differ significantly (*p* = n.s.).

There were significant differences between the genders as shown in Table 3. In general, females presented significantly higher femoral internal torsion and a higher mMPTA than males. Overall females had minimal valgus alignment, while males presented slight varus alignment. The age, tibial torsion, mLPFA, mLDFA and mLDTA did not differ significantly between genders.

**Table 3:** Age, coronal and torsional parameters by gender; measured parameters are displayed as mean and SD.

	Male	Female	T value	*p* Value	Mean difference	95% CI
Age (years)	33.6 (14.4)	34.5 (13.8)	−0.64	n.s.	−0.9	−3.9–2.0
Femoral torsion (°)	−25.8 (10.8)	−30.9 (10.2)	4.58	**<0.001**	5.1	2.9–7.3
Tibial torsion (°)	35.0 (8.5)	34.9 (9.7)	0.04	n.s.	0.1	−1.9–1.9
mFA‐mTA (°)	1.3 (3.3)	−0.2 (2.9)	4.49	**<0.001**	1.5	0.8–2.1
mLPFA (°)	88.3 (5.2)	88.4 (5.6)	−0.19	n.s.	−0.11	−1.2–1.0
mLDFA (°)	87.6 (2.4)	88.0 (2.6)	−1.62	n.s.	−0.44	−0.9–0.1
mMPTA (°)	86.9 (2.4)	88.7 (2.1)	−7.1	**<0.001**	−1.68	−2.2 to −1.2
mLDTA	86.9 (4.6)	86.9 (4.3)	‐	n.s.	‐	‐

*Note*: Bold values represent statistically significant results.

Abbreviations: CI, confidence interval; mFA‐mTA, mechanical tibiofemoral angle; mLDFA, mechanical lateral distal femoral angle; mLDTA, mechanical lateral distal tibial angle; mLPFA, mechanical lateral proximal femoral angle; mMPTA, mechanical medial proximal tibial angle; n.s., not significant; SD, standard deviation.

**Figure 3:**
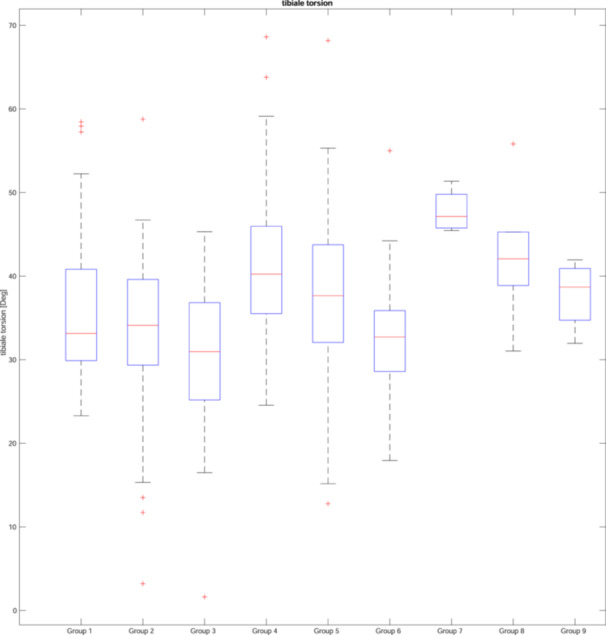
Tibial torsion and knee phenotypes according to coronal plane alignment of the knee classification.

**Figure 4:**
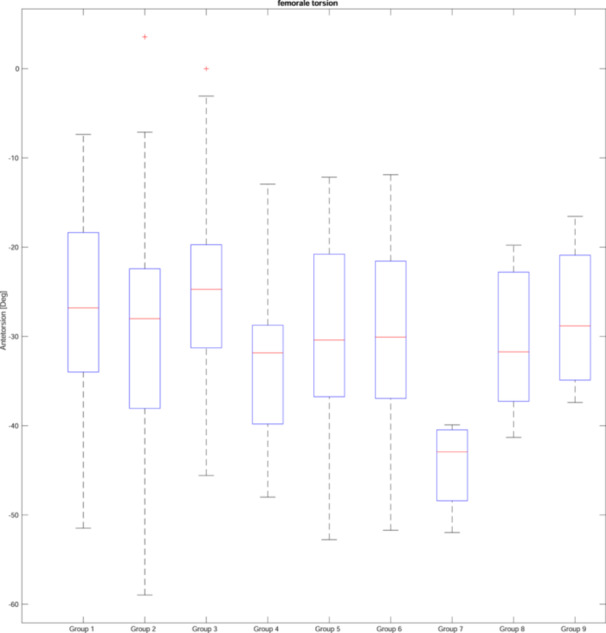
Femoral torsion and knee phenotypes according to coronal plane alignment of the knee classification.

**Figure 5:**
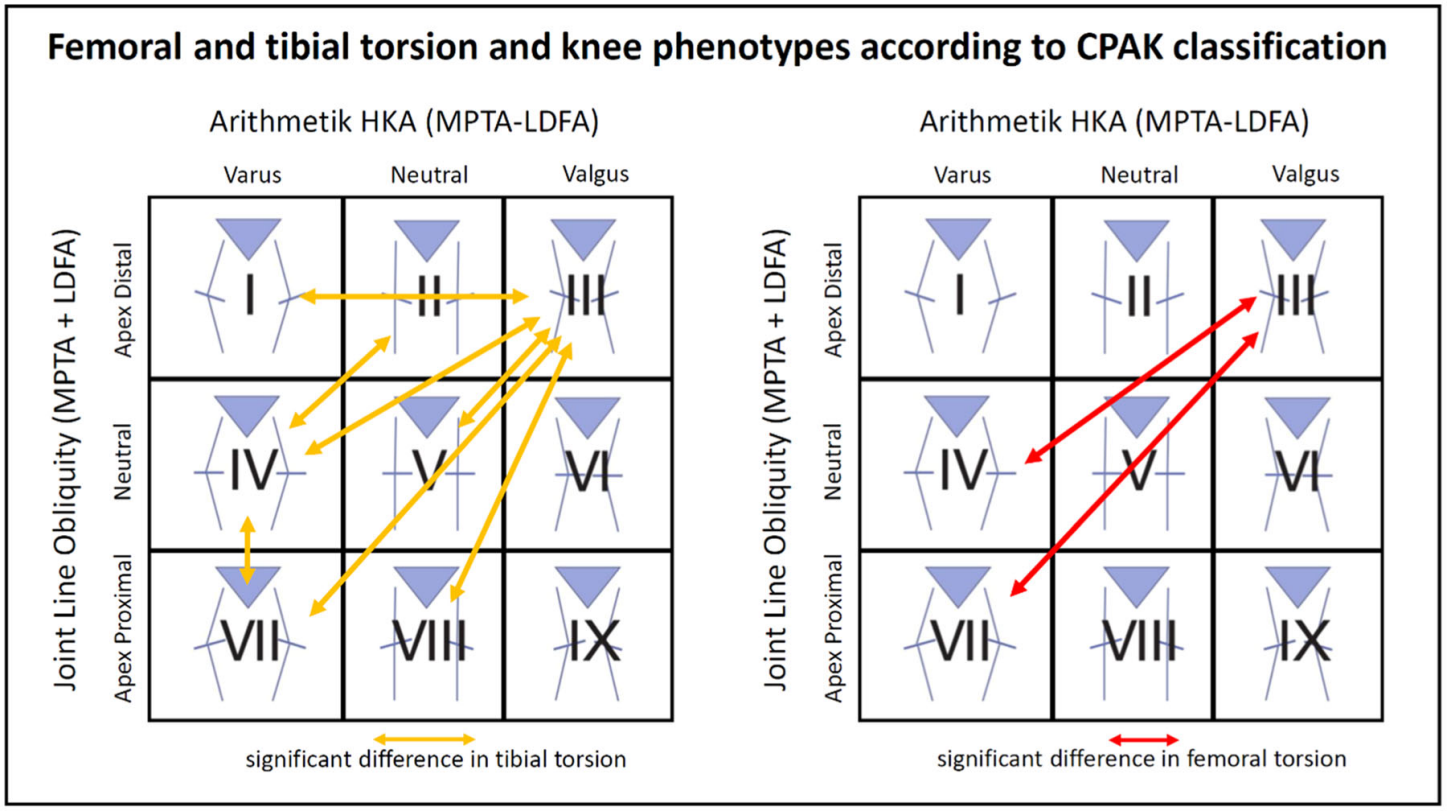
Femoral and tibial torsion and knee phenotypes according to coronal plane alignment of the knee classification.

Femoral and tibial torsion were also analyzed according to the CPAK classification [21], displayed in Figures 3 and 4. The conducted ANOVA showed significant differences between groups for femoral as well as tibial torsion (both *p* < 0.001). Alpha‐adjusted post hoc Bonferroni tests showed that significant differences could be found for femoral torsion between CPAK groups III–IV (*p* = 0.010) and groups III–VII (*p* = 0.008). For tibial torsion significant differences could be showed between CPAK groups I–III (*p* = 0.029), groups II–IV (*p* = 0.001) and groups III–IV (*p* = 0.000), groups III–V (*p* = 0.001), groups III–VII (*p* = 0.005), groups III–VIII (*p* = 0.046), group IV–VI (*p* = 0.010). Figure 5 displays the results graphically.

## DISCUSSION

The most important findings of this study were that the coronal alignment does not enable to predict the degree of femoral and tibial torsion, but the tibial external torsion increased as the coronal alignment changed from valgus to varus and the torsional parameters both increased with higher mLDFA. In addition, as the coronal alignment changed from valgus the femoral internal torsion increased in the male group. These results are also reflected in the results concerning the CPAK classification [31].

The results of studies on leg alignment are highly influenced by the demographic parameters of their study population and the applied measurement methods. Both ethnicity and gender might influence the alignment in the frontal and axial planes. Asians tend to have reduced tibial torsion [14, 22], higher femoral internal torsion [22] and more frequent varus alignment [14] than Caucasians. Meanwhile, females tend to have higher femoral internal torsion [22, 27] and more frequent valgus alignment than males [1, 4]. Therefore, while comparing studies on the lower extremity alignment, their demographic data must be taken into consideration.

Existing studies on the relationship between coronal and axial alignment of the lower leg alignment showed inconsistent results. Chang et al. [3] found a higher femoral internal torsion in their valgus group than in their neutral and varus group, when using the posterior condylar line as the distal femoral torsional axis. In accordance with that, the femoral internal torsion increased, as the coronal alignment changed from varus to valgus, but the correlation was fairly weak (*r* = 0.145). However, their study showed no significant correlation between the femoral torsion and the coronal alignment, if the clinical transepicondylar axis was used as the distal femoral torsional axis [3].

Other studies did not find any correlation between the femoral torsion and the coronal alignment [20, 23]. These findings do not agree with the results of the present study. In this study population, the femoral internal torsion increased as the coronal alignment changed from valgus to varus. However, subgroup analysis showed that this result was limited to the male group and, therefore, seems to be gender‐related. Femoral internal torsion did not differ significantly between the valgus, neutral and varus groups in the female population.

Chang et al. [3] and Leon‐Munoz et al. [20] both found higher tibial external torsion in their valgus group than in their varus group and reported a linear correlation between the tibial torsion and the coronal alignment: as the coronal alignment changed from varus to valgus, tibial external torsion increased. On the other hand, the studies by Nejima et al. [23] and Lee et al. [19] presented no significant relations between the tibial torsion and coronal alignment.

Contrary to that, the present study found the highest tibial torsion in the varus group as well as an increasing tibial torsion as the coronal alignment changed from valgus to varus.

To the best of our knowledge, Nejima et al. were the first to examine whether there is a correlation between the joint angles and the torsional profile of the lower extremity. Their analysis was limited to the mLDFA and mMPTA. They found no relationship between the torsional parameters and the mLDFA but reported an increasing femoral internal and tibial external torsion with lower mMPTA. The present study was not able to confirm a relationship between the mMPTA and the femoral and tibial torsion. Yet we found that the femoral internal and tibial external torsion increased with higher mLDFA.

The differences in the described results might exist due to demographic differences. All the compared studies examined patients with OA only. The study populations of Chang et al. [3], Nejima et al. [23] and Lee et al. [19] included only Asian patients with female predominance. Leon‐Munoz et al. [20] analyzed the leg alignment of Caucasian patients, but their valgus group was very small compared to their varus group (53 valgus knees vs. 251 varus knees). The same is true for the group sizes in the study by Chang et al. [3] (31 valgus knees vs. 313 varus knees).

Taking all this into consideration the present study is the most reliable in terms of group distribution (55.3% females; 113 valgus knees vs. 128 varus knees) and, therefore, gives a good overview of the lower leg alignment in a German population with little to no knee OA.

In accordance with works by other authors [20, 22, 27], females presented with significantly higher femoral internal torsion than males in this study. There was no gender‐specific difference in terms of the tibial torsion, which supports the results by Mathon et al. [22] and Imhoff et al. [15]. However, Leon‐Munoz et al. [20] described significantly higher tibial torsion in their female population in comparison to males. This discrepancy might be based on the vast range of tibial torsion in the general population.

There are some limitations to our study: measurements were taken only once by a single observer. The analysis of the leg alignment did not include the sagittal plane. Hence an influence of the lower extremity alignment in the sagittal plane on the femoral and tibial torsion cannot be ruled out.

## CONCLUSION

Even though the present study established the trend that the tibial torsion increased with varus alignment and that the femoral and tibial torsion are related to the mLDFA, the correlations were weak. The coronal alignment does not enable to predict the degree of femoral and tibial torsion. Hence, this study demonstrates that an individual approach to each patient with malalignment is unavoidable.

## AUTHOR CONTRIBUTIONS

**Leonard Grünwald**: Conceptualization (equal) and study protocol (lead); creating the database (lead); literature research (equal); additional statistics (lead); review and editing (equal). **Sophie Schmidt**: Writing—original draft (lead); formal analysis (lead); literature research (equal); writing—review and editing (equal). **Marc‐Daniel Ahrend**: Writing—review and editing (equal). **Tina Histing**: Conceptualization (lead); review and editing (equal); Supervision (lead). **Stefan Döbele**: Writing—review and editing (equal); project administration (lead).

## CONFLICT OF INTEREST STATEMENT

The authors declare no conflict of interest.

## ETHICS STATEMENT

All procedures performed in the study involving human participants were in accordance with the ethical standards of the institutional and/or national research committee and with the 1964 Helsinki Declaration and its later amendments or comparable ethical standards. Institutional review board approval about all aspects of the study from an ethical and legal point of view was obtained (IRB number 695/2020B02). Due to the retrospective approach, no patient contact occurred in this study and informed consent was not needed to be obtained.

## Supporting information

Supplementary Information

## Data Availability

The data that support the findings of this study are available from the corresponding author, (S. D.), upon reasonable request.
